# Individual differences in satisfaction with activity-based work environments

**DOI:** 10.1371/journal.pone.0193878

**Published:** 2018-03-08

**Authors:** Jan Gerard Hoendervanger, Anja F. Ernst, Casper J. Albers, Mark P. Mobach, Nico W. Van Yperen

**Affiliations:** 1 Hanze University of Applied Sciences, Groningen, The Netherlands; 2 University of Groningen, Groningen, The Netherlands; 3 The Hague University of Applied Sciences, The Hague, The Netherlands; University at Buffalo - The State University of New York, UNITED STATES

## Abstract

Satisfaction with activity-based work environments (ABW environments) often falls short of expectations, with striking differences among individual workers. A better understanding of these differences may provide clues for optimising satisfaction with ABW environments and associated organisational outcomes. The current study was designed to examine how specific psychological needs, job characteristics, and demographic variables relate to satisfaction with ABW environments. Survey data collected at seven organizations in the Netherlands (*N* = 551) were examined using correlation and regression analyses. Significant correlates of satisfaction with ABW environments were found: need for relatedness (positive), need for privacy (negative), job autonomy (positive), social interaction (positive), internal mobility (positive), and age (negative). Need for privacy appeared to be a powerful predictor of individual differences in satisfaction with ABW environments. These findings underline the importance of providing work environments that allow for different work styles, in alignment with different psychological need strengths, job characteristics, and demographic variables. Improving privacy, especially for older workers and for workers high in need for privacy, seems key to optimizing satisfaction with ABW environments.

## Introduction

In order to cut costs and support contemporary knowledge work, a growing number of organizations is adopting a work environment based on the principles of activity-based working (ABW) [1–3]. In ABW environments, workers do not have assigned workstations but instead share an area with different types of non-assigned ‘activity settings’, e.g., workstations in open-plan areas and in closed rooms, open meeting spaces, and closed meeting rooms. These different settings are designed for different types of activities [4–6], e.g., collaboration, concentration, communication, creativity, confidentiality, and contemplation [7]. Because there are fewer workstations than workers in ABW environments, less office space is needed and hence cost-cutting targets are usually met [8]. However, when it comes to supporting the work, the effectiveness of ABW environments is not evident.

So far, empirical findings have shown that satisfaction with ABW environments is usually below expectations, with concentration, privacy, and the loss of an assigned workstation major self-reported issues [9–11]. Optimizing satisfaction with the physical work environment is important for organizations, since this has been found to be related directly to job satisfaction and indirectly to other organizational outcomes such as commitment, intention to leave, engagement, and absenteeism [12–15]. The costs associated with decreased commitment, increased staff turnover, and increased absenteism may easily exceed the savings regarding real estate, as these latter costs typically comprise less than 10% of the total costs of knowledge work [16,17]. Hence, dissatisfaction with the work environment may not only be an indicator of sub-optimal support of knowledge work, but also a threat to the objective to cut costs.

Searching for factors that may explain differences in satisfaction with ABW environments, and hence provide clues for optimizing associated organizational outcomes, Brunia et al. [18] compared four case studies conducted at four different branches of one organization. They found large differences in satisfaction with the work environment that were mainly related to interior design, level of openness, subdivision of space, number and diversity of workplaces, and accessibility of the building. Furthermore, satisfaction with the work environment appeared to be associated with the process of implementing it and with satisfaction with the organization in general. Besides these situational differences, large differences in satisfaction with the work environment have also been observed among individual workers who share the same ABW environment [19]: Wohlers & Hertel suggest that both person-related and job-related variables may play a role in these differences [2]. Several scholars have addressed the importance of individual differences with regard to perception of work environments (e.g., [9,20–22]). According to Person-Environment fit (PE fit) theory, dissatisfaction may result from a misfit between certain psychological needs and environmental conditions [23,24]. In this respect, empirical studies have shown the relevance of need for autonomy, need for relatedness, and need for structure in relation to flexible working across different locations [25], and of need for privacy in relation to workspace layout [26]. These findings suggest that these particular psychological needs may be relevant for achieving PE fit in ABW environments. However, similar studies within ABW contexts have not yet been conducted. Since the concept of ABW was developed with specific job characteristics in mind–i.e., high job autonomy, high social interaction, and high internal mobility [27,28]–it seems likely that these job characteristics are positively related to satisfaction with ABW environments. However, these assumed relationships have not yet been tested in empirical research. With regard to demographic variables, age and gender seem relevant. Empirical findings indicate that age may be negatively associated with satisfaction with ABW environments [29], while a positive relationship might be expected based on the literature on age-related differences in the workplace [2]. Although men and women seem to experience certain aspects of ABW environments differently [2], they have been found to be equally satisfied with ABW environments [3].

Given these research gaps, the current study was designed to empirically examine how satisfaction with ABW environments is associated with the aforementioned psychological needs (i.e., need for autonomy, relatedness, structure, and privacy), job characteristics (i.e., job autonomy, social interaction, and internal mobility), and demographic variables (i.e., gender and age). The first aim was to test literature-based hypotheses for each of these independent variables. The second aim was to find out which of these variables are useful for predicting individual differences in satisfaction with ABW environments. In explaining such differences, this study may contribute to the furthering of PE fit theory. Its practical relevance concerns the optimization of satisfaction with work environments and associated organizational outcomes.

## Psychological needs

We included four basic psychological needs that seem particularly relevant in the context of ABW environments: (1) need for autonomy, (2) need for relatedness, (3) need for structure, and (4) need for privacy. In line with PE fit theory [23,24] and other organizational theories that recognize the importance of individual differences (e.g., [30,31]), we focused on psychological needs strength. According to PE fit theory, workers high in need for X will be more satisfied with a work environment providing X, and less satisfied with a work environment lacking X, compared with workers low in need for X.

Need for autonomy is defined as workers’ *“desire to feel volitional and to experience a sense of choice and psychological freedom when carrying out an activity”* ([32], p.982). ABW environments provide a high level of autonomy because workers have freedom of choice regarding the use of different work settings, instead of being assigned to one specific workstation [2]. Van Yperen et al. [25,33] demonstrated that workers high in need for autonomy preferred location-independent working, i.e., flexibility in where work gets done across different locations (including the office). In line with these findings, we expected workers high in need for autonomy to prefer flexibility in where work gets done *within* the office. Hence, we hypothesized (*Hypothesis 1*) that workers’ need for autonomy would be *positively* associated with satisfaction with ABW environments.

Need for relatedness, defined as workers’ *“propensity to feel connected to others*, *that is*, *to be a member of a group”* ([32], pp. 982–983), requires *“frequent*, *affectively pleasant or positive interactions with the same individuals (…) in a framework of long-term*, *stable caring and concern”* ([34], p. 520). ABW environments are usually designed to promote social interaction through openness, transparency, and informal meeting spaces [2]. As a consequence, ABW environments should provide ample opportunity to build and maintain strong relationships with colleagues. Accordingly, we hypothesized (*Hypothesis 2*) that workers’ need for relatedness would be *positively* associated with satisfaction with ABW environments.

Need for structure is defined as a workers’ *“need for clarity and intolerance of ambiguity”* and refers to a *“preference for structured and predictable situations”* ([35], p. 23). Workers high in need for structure may dislike the fact that uncertainty about the availability of specific work settings at specific moments is inherent to the ABW concept, as this may create ambiguity. Van Yperen et al. [25,33] demonstrated that–probably for the same reason–workers low in need for structure preferred blended working (i.e., time-independent and location-independent working). In the current study, we hypothesized (*Hypothesis 3*) that workers’ need for structure would be *negatively* associated with satisfaction with ABW environments.

Need for Privacy, defined as a worker’s *“need for physical isolation from stimuli”* ([26], p. 255), refers to architectural privacy (i.e., visual and acoustic isolation) that contributes to psychological privacy (i.e., sense of control over access to oneself) [36]. Altman [37] proposed that people try to achieve an optimal level of privacy, in accordance with their needs and circumstances, by seeking or avoiding social interaction. In an empirical study, a high need for privacy was found to be associated with a preference for working in enclosed instead of open workspaces [26]. Although ABW environments typically provide both open and enclosed workspaces, the design is usually predominantly open and transparent [2]. Previous research indicates that an experienced lack of privacy may be one of the major complaints underlying low levels of satisfaction with ABW environments [9,11]. Therefore, we hypothesized (*Hypothesis 4*) that workers’ need for privacy would be *negatively* associated with satisfaction with ABW environments.

## Job characteristics

We included three job characteristics on which the ABW concept is grounded: (1) job autonomy, (2) social interaction, and (3) internal mobility. According to Davenport [38], these job characteristics are typical of contemporary knowledge workers. Duffy and Powell [27] theorized that an ABW environment (a ‘club’ in their terminology) would be particularly suitable for ‘transactional knowledge work’, i.e., work that is characterized by a high level of job autonomy and a high level of social interaction. In their view, workers in this type of job should be enabled to move freely and frequently across the work environment, alternating the use of settings for solo work and settings for meetings and collaborative tasks.

Job autonomy refers to the degree of control of an employee over how to carry out the job task [39]. In an ABW context, this particularly includes choices regarding the use of different places for different activities. Since ABW environments typically provide a range of choices of different settings for different activities, which can be freely used on an as-needed basis [4–7], we may expect a good fit with high job autonomy. Hence, we hypothesized (*Hypothesis 5*) that job autonomy would be *positively* associated with satisfaction with ABW environments.

Research on knowledge sharing has shown the importance of unplanned face-to-face meetings for sharing tacit knowledge, and how spatial variables (i.e., visibility, proximity) may facilitate such meetings [40]. In line with these insights, ABW environments are usually designed to promote social interaction through openness, transparency, and informal meeting spaces [2]. Accordingly, we hypothesized (*Hypothesis 6*) that workers’ perceptions that social interactions are part of their jobs would be *positively* associated with satisfaction with ABW environments.

Mobility within the work environment is directly related to the basic idea underlying the ABW concept, as workers need to move around to be able to use different types of activity settings for different types of activities [6,28]. This internal mobility may typically go hand in hand with social interactions across the office building [41]. Since ABW environments facilitate internal mobility, a good fit–resulting in satisfaction with the work environment–may be expected with jobs that require workers to move frequently between different workplaces. A recent empirical study indeed found a positive relationship between switching frequency and satisfaction with ABW environments [19]. Hence, we hypothesized (*Hypothesis 7*) that workers’ perceptions that internal mobility is a characteristic of their jobs would be *positively* associated with satisfaction with ABW environments.

## Demographic variables

Two basic demographic variables were examined in this study: age and gender. It has been found that older workers report lower satisfaction ratings with ABW environments than younger workers [3,29,42]. According to Pullen [29], this might be related to an increased sensitivity to auditory and visual distractions at greater ages, as observed in several studies (e.g., [43,44]). This seems to be confirmed by his data: older workers are negative about the openness and transparency of ABW environments compared with cell offices, whereas younger workers regard this difference positively [29]. Hence, we hypothesized (*Hypothesis 8*) that age would be *negativel*y associated with satisfaction with ABW environments.

According to Wohlers and Hertel [2], men and women may experience certain aspects of ABW environments differently. As women are more inclined to personalize their workspaces [45], which is typically not allowed in ABW environments, they might be less satisfied than men. Also, women have been found to complain more about noise distractions in open-plan environments than men [42,46]. On the other hand, it has been observed that men complain more about desk-sharing than women [42]. A recent study by Leesman [3] found no significant differences between men and women regarding their satisfaction with ABW environments. Since previous findings regarding possible gender differences were inconclusive, we included this variable in the current study without relating it to a hypothesis.

## Method

### Ethics statement

This research was reviewed and approved by the ethics committee of the Department of Psychology, University of Groningen. The data were analyzed anonymously.

### Sample

The current study was cross-sectional; an online questionnaire was administered to employees once. Data were collected in seven Dutch organizations, found within the researchers' networks, that agreed to cooperate by allowing their employees to take part in the survey. The researchers monitored the data collection process and inspected the work environments of the participating organizations. All work environments were based on the ABW concept, offering a variety of non-assigned activity settings, including a main area with workstations in an open-plan layout and (semi-)enclosed back-up spaces for meetings, concentration work, and phone calls. Apart from these similarities, there were also situational differences between the seven organizations (e.g., interior design, work processes, organizational culture, implementation processes) that were beyond the scope of this study. We statistically controlled for these situational differences by including organizational affiliation in the regression analysis.

The total sample comprised data provided by 551 knowledge workers at seven different organizations. These organizations operate in different parts of the private and public sector, and are located in different Dutch cities. Descriptive information about the organizations is presented in Table 1. The large sample size ensures high power: *N* = 551 gives a power of .8 to find a significant effect at the *α* = .05 level for a correlation of *r* = .12. Examination of the residuals of the final regression model showed no violations of independence, normality, homogeneity, or linearity assumptions (S2 Appendix).

**Table 1:** Descriptive information about the included organizations.

*Organization*	*Organization type*	*Location*	*n*	*Age*				*% male*
				*Mean*	*SD*	*Min*.	*Max*.	
1.	Public organization	Groningen	168	49.69	9.60	22	64	60%
2.	Private company	Groningen	53	40.92	9.52	23	63	74%
3.	Public organization	Assen	117	43.04	9.92	28	60	43%
4.	Educational organization	Utrecht	23	47.19	10.26	25	64	46%
5.	Public service provider	Multiple locations	108	44.56	10.33	25	63	54%
6.	Private company	Amsterdam	50	37.22	7.78	23	68	34%
7.	Private company	Amsterdam	32	45.25	9.62	29	60	28%
Total sample			551	45.64	10.43	22	68	52%

Table 1 presents organization type, location, sample size, age structure, and gender distribution for the included organizations.

### Measures

Satisfaction with the work environment was assessed using a single item with response values ranging from one to ten, also allowing quarter values, with lower values corresponding to lower satisfaction. We consider use of a single item acceptable because workplace satisfaction is a sufficiently narrow and unambiguous construct [47].

Psychological needs for autonomy, relatedness, and structure were assessed using the questionnaire developed by Van Yperen, Rietzschel, and De Jonge [25], drawing on existing measures used to assess need satisfaction (autonomy and relatedness) rather than need strength [32] and personal need for structure [48]. It includes questions such as: “I need to have a say in determining my activities and tasks” (need for autonomy), “I have the need to feel like I am part of a team or a group” (need for relatedness), and “I have the need for a daily routine” (need for structure). The reliabilities of all subscales were high, with Cronbach's alphas of .89 for need for autonomy, .86 for need for relatedness, and .80 for need for structure.

Need for privacy was determined using a subsection of the Environmental Response Inventory [49,50], which yielded a Cronbach's alpha of .84 on our sample. The scale consists of four questions, such as, “I get distracted easily”.

Job characteristics were assessed using one statement for each job characteristic. These were: (1) “I have freedom to carry out my tasks the way I prefer” (job autonomy); (2) “My work involves interaction with other people” (social interaction); (3) “I work at different places within the office building” (internal mobility). We consider use of single items acceptable because these job characteristics are sufficiently narrow and unambiguous constructs [47].

All psychological needs and job characteristics variables were scored on a scale with values from one (“very strongly disagree”) to five (“very strongly agree”), allowing quarter values (i.e., possible values increased in a .25-step fashion). The complete questionnaire is provided as supporting information, S1 Appendix.

## Results

### Descriptive statistics

In Table 2, descriptive statistics are presented for all included continuous variables. Satisfaction with the work environment was rated 6.9 on average, with individual ratings ranging from 1 till 10. Further examination of these ratings showed that 84% of all respondents rated their satisfaction level as 5.5 or higher. This means that this group may be regarded as (more or less) satisfied according to the grading system that is commonly used in schools in the Netherlands, in which a (rounded) 6 defines the distinction between “pass” and “fail”.

**Table 2:** Descriptive statistics for the included continuous variables.

*Variable*	*Mean*	*SD*	*1*.	*2*.	*3*.	*4*.	*5*.	*6*.	*7*.	*8*.
1. Satisfaction with the work environment	6.87	1.57	-							
2. Need for autonomy	3.83	.73	.05	-						
3. Need for relatedness	3.01	.74	.10*	.04	-					
4. Need for structure	2.52	.71	-.07	-.27***	.12**	-				
5. Need for privacy	2.52	.79	-.39***	.06	-.13**	.27***	-			
6. Job autonomy	3.87	.79	.21***	.51***	.04	-.23***	-.09*	-		
7. Social interaction	3.92	.82	.22***	.40***	.18***	-.19***	-.15***	.40***	-	
8. Internal mobility	2.54	1.12	.14***	.21***	.03	-.24***	-.01	.30***	.25***	-
9. Age	45.64	10.43	-.21***	-.04	-.22***	-.07	-.01	-.01	-.11**	-.19***

Table 2 presents for each continuous variable the mean, standard deviation, and Pearson product-moment correlations with the other variables (column labels 1–8 refer to the corresponding variables in rows 1–8); * *p* < .05; **p < .01; *** *p* < .001. S1 Table provides 95% confidence intervals and exact *p*-values for the correlations.

The correlation coefficients in Table 2 (column “1.”–“8.”) indicate how the variables are related to each other. Need for privacy comes to the fore as the strongest correlate of satisfaction with the work environment.

Our sample contains 52% male and 48% female workers. The results of a two-sided *t*-test suggest that there is no significant gender difference in satisfaction with ABW environments (*t* = 1.80, *df* = 549, *p* = .07).

### Hypothesis testing

The correlation coefficients (see Table 2, column “1.”) indicate that empirical support was found for six of our hypotheses: satisfaction with ABW environments is positively and significantly associated with need for relatedness (*Hypothesis 2*), job autonomy (*Hypothesis 5*), social interaction (*Hypothesis 6*), and internal mobility (*Hypothesis 7*), and it is negatively and significantly associated with need for privacy (*Hypothesis 4*) and age (*Hypothesis 8*). According to the correlation coefficients, the effect sizes of the associations can be regarded as ‘medium to large’ for need for privacy, ‘small’ for need for relatedness, and ‘small to medium’ for the other significant associations [51]. No empirical support was obtained for our hypotheses regarding need for autonomy (*Hypothesis 1*) and need for structure (*Hypothesis 3*).

### Regression analysis

To identify useful predictors of satisfaction with ABW environments, a regression model was developed using a stepwise forward model selection procedure. The control variable organizational affiliation was constrained to be included in the minimal model after an ANOVA *F*-test showed that satisfaction with the work environment differed significantly between the organizations in our sample (*F* = 22.24, *df* = 544, *p* < .001). Other variables were added to this minimal model one by one. At each step the variable was added which explained the most variance in the outcome variable that was still left unexplained by the variables already included in the model. This forward selection was terminated when none of the remaining variables improved the model fit to a substantial extent, as indicated by the Bayesian Information Criterion (BIC). The BIC is an indicator of model fit that penalizes the inclusion of parameters, thereby favoring more simplistic models with fewer parameters [52]. Consequently, predictors will only be selected to the final model if they are associated with a considerable increase in model fit, and the final model will consist of only the most powerful predictors of satisfaction with ABW environments. The order in which variables were added to the minimal model, which included only organizational affiliation as predictor, is shown in Table 3. Through the increase in adjusted *R*^*2*^ this table indicates the amount of remaining variance the newly added variable explains over and above what is already explained by the variables already included in the model at that stage. Thus, the stepwise selection procedure identified a regression model that includes organizational affiliation, need for privacy, age, job autonomy, and need for autonomy as the ideal model. The results of this model are summarized in Table 4.

**Table 3:** Steps in the model selection procedure.

*Predictor added to the regression model*	*BIC of the regression model after each step*	*Adjusted R*^*2*^ of the regression model after each step
Start: Organizational affiliation (minimal model)	423.99	.18
Step 1: Need for Privacy	348.48	.29
Step 2: Job autonomy	343.64	.30
Step 3: Age	341.28	.32
Step 4: Need for autonomy	340.17	.32

**Table 4:** Results of the final regression model.

	*Standard Error*	*Estimate*	*95% confidence interval*	*p-value*
*β*_*0*_ (Intercept)	.47	8.00	7.07–8.92	> .001
*β*_Organisation 2_	.21	1.36	.94–1.78	> .001
*β*_Organisation 3_	.30	1.19	.62–1.77	> .001
*β*_Organisation 4_	.16	.84	.52–1.16	> .001
*β*_Organisation 5_	.17	1.31	.98–1.64	> .001
*β*_Organisation 6_	.23	1.28	.83–1.72	> .001
*β*_Organisation 7_	.26	1.28	.78–1.78	> .001
*β*_Need for privacy_	.07	-.64	-.78 - -.49	> .001
*β*_*J*ob autonomy_	.08	.36	.19 - .52	> .001
*β*_Age_	.01	-.02	-.03 - -.01	.004
*β*_Need for autonomy_	.09	-.25	-.43 - -.07	.007

Table 3 presents the increase in model fit after each variable was added in a stepwise fashion in the model selection procedure. In each step the variable that explained the most of the remaining variance in the outcome variable was added to the regression model.

Table 4 presents standard errors, estimates, confidence intervals, and *p*-values of the final regression model; organization 1 is the baseline with which other organizations are compared.

The final model explains 32% of the variance in satisfaction with ABW environments (*R*^*2*^_*Adj*_ *=* .*32*). As the stepwise increases in *R*^*2*^ in Table 3 indicate, the lion’s share of this percentage is contributed by organizational affiliation and need for privacy. After need for privacy was included, age, job autonomy, and need for autonomy added marginal predictive power to the model. Hence, the regression results indicate that, for a given organization, workers’ need for privacy may be used to predict their satisfaction with the ABW environment.

Job autonomy is the only job characteristic that was included in the final model; social interaction and internal mobility were also significantly correlated with satisfaction with the work environment. This implies that social interaction and internal mobility do not explain a significant proportion of variance in satisfaction with ABW environments over and above the variance explained by job autonomy; this may be due to significant mutual correlations (see Table 2). Similarly, need for relatedness, although correlated with satisfaction with the work environment, did not additionally explain any variance.

The inclusion of need for autonomy in the final model as a (weak) negative predictor was unexpected, both in relation to our hypothesized positive association (*Hypothesis 1*) and in relation to its non-significant correlation with satisfaction with the work environment (see Table 2). Apparently, need for autonomy added marginal predictive power to the model due to its relations with the other predictor variables.

Residuals of our final regression model were examined closely for violation of the independence, normality, and linearity assumptions. No violation of regression assumptions was detected, indicating that model errors are uncorrelated between employees even though employees were sampled nested within organizations. Diagnostic plots of our model residuals can be found in the supporting material, S2 Appendix.

## Discussion

### Interpretation and explanation of the results

This study identified two psychological needs, three job characteristics, and one demographic variable as significant correlates of satisfaction with ABW environments: need for relatedness (positive), need for privacy (negative), job autonomy (positive), social interaction (positive), internal mobility (positive), and age (negative). No empirical support was found for the hypothesized associations with need for autonomy and need for structure.

Need for privacy appeared to be a powerful predictor of individual differences in satisfaction with ABW environments.

Although weak and barely significant (*r* = .11, p < .05), the correlation between need for relatedness and satisfaction with ABW environments indicates that the openness, transparency, and informal meeting spaces typical of ABW environments [2] are valued by workers who focus on social interactions.

The strong association with need for privacy clearly confirms previous findings indicating that ABW environments fail to provide satisfactory levels of privacy [9,11]. This shows an important downside of the aforementioned openness and transparency. The work environments included in this study all feature a main area with workstations in an open-plan layout, providing low levels of acoustic and visual privacy. Especially since most workers switch seldom or not at all between different activity settings [19], they may frequently find themselves in these open-plan areas, also when performing activities that require concentration. Apparently, the enclosed quiet spaces for concentration work, which were present in all work environments as well, do not resolve privacy issues adequately.

Need for autonomy did not show a significant correlation with satisfaction with ABW environments; however, it was included in the final regression model as a weak negative predictor of satisfaction with ABW environments. Apparently, the range of choices of different activity settings falls short of fulfilling the need for autonomy of those workers who strongly feel this need. Referring to a study by Gerdenitsch et al. [53], this may indicate that the freedom of choice in ABW environments is perceived as not only autonomously but also externally controlled. In this context, perceived external control might be related to social barriers (e.g., social norms) and practical barriers (e.g., limited availability of preferred settings) to using different activity settings freely. Also, not having any personal territory within the work environment might lead to a perception of external control.

Need for structure does not seem to be associated with satisfaction with ABW environments. Uncertainty about the availability of specific work settings at specific moments does not seem to harm workers’ sense of structure. This finding might be explained by the aforementioned low switching frequencies [19]; workers who mostly or always use the same activity setting may not actually experience uncertainty regarding their use.

Workers who perceived their jobs as providing a high degree of autonomy (i.e., freedom to choose when, where, and how to work) reported higher satisfaction ratings. A possible explanation is that this freedom enables workers to benefit from the opportunity offered by an ABW environment to choose an activity setting that fits their needs at all times. We might also expect these workers to avoid certain drawbacks of ABW environments (e.g., lack of privacy, shortage of favorite activity settings) by working at alternative locations (e.g., at home, co-working space) whenever they like.

In line with our hypothesis, workers in jobs that require a high degree of social interaction and workers in jobs that require a high degree of internal mobility expressed higher levels of satisfaction with ABW environments. This seems to indicate that ABW environments support job-related social interactions and internal mobility.

The observed negative association between age and satisfaction with ABW environments indicates, in accordance with Pullen [29] and Leesman [3], that these environments are less well suited to the needs and preferences of older workers. Our finding regarding need for privacy seems to confirm the idea, put forward by Pullen [29], that this might be due to an increased sensitivity to auditory and visual distractions at greater ages. In addition, our finding regarding age seems to match with the observation that older knowledge workers particularly miss quiet spaces to contemplate and recuperate [54]. Our finding regarding age contradicts the expectations of Wohlers and Hertel [2], based on the more active coping strategies and better self-regulation skills found in older workers compared with younger workers. Apparently, these better developed skills are not helping older workers sufficiently to resolve their problems with concentration and privacy in ABW environments.

In line with previous research by Leesman [3], we found male and female workers to be equally satisfied with ABW environments. This may indicate that gender-based affective differences regarding certain aspects of ABW environments, as mentioned by Wohlers and Hertel [2], do not greatly affect overall satisfaction, possibly because male and female complaints (regarding different aspects) balance each other out.

### Limitations and perspectives for further research

The size of our data set, and its combination of data from seven different organizations, enabled us to draw clear conclusions about factors associated with individual differences in satisfaction with ABW environments. Since ABW is an international trend [1–3], it would be interesting to confirm the international relevance of our findings in further research, by including data from organizations in different countries.

Although any survey-based study, like the current one, is limited with regard to the number of factors that can be included, the search for factors that may explain individual differences in satisfaction with ABW environments might be broadened in future research. For instance, certain personality traits (e.g., Big Five dimensions) might be included to enable replication of previous findings in this field (e.g., [20]). Also, more job-related variables might be included in further research, such as aspects of activity profiles (e.g., share of office time spent on different types of activities). As the heterogeneity of activity profiles (i.e., dispersion of office time over different types of activities) was found to be related to switching behavior [19], it might also affect satisfaction with ABW environments.

Our main finding regarding the need for privacy stresses the need for further research focusing on why workers, and especially older workers and workers high in need for privacy, experience a lack of privacy in ABW environments and how this issue might be resolved. Zooming in on this complicated matter, which seems to involve an interplay of multiple functional factors (e.g., architectural privacy, availability of settings for concentrated work), physiological factors (e.g., increased sensitivity to auditory and visual stimuli), psychological factors (e.g., need for privacy, switching behavior), and social factors (e.g., social interaction and social norms), probably requires a qualitative research design, using techniques like close observations and in-depth interviews.

Many of our findings, including non-significant relationships, seem to be associated with workers’ choice behaviors and switching behaviors in ABW environments. Further research might explain (individual differences regarding) these behaviors and their impacts on satisfaction with ABW environments. Thorough examination of behavioral patterns requires advanced measurement techniques (e.g., experience sampling, sensor-based location tracking), which do not rely on retrospective recall of these patterns.

Although it was beyond the scope of the current study, our results strongly confirm that satisfaction with ABW environments may differ widely across organizations. Highly significant differences were found in our sample, with a 1.36 difference on average (on a ten-point scale) between the organization with the highest ratings and the organization with the lowest ratings. Compared with a different dataset that was previously used by Hoendervanger et al. [13], the average satisfaction score in the current sample was high (6.9 vs. 5.6), with a greater share of workers that may be regarded as satisfied (84% vs. 60%). These striking differences call for further research examining situational differences, in line with the comparative case study by Brunia et al. [18].

### Implications for theory and practice

We believe that our findings are relevant to Person-Environment fit (PE fit) theory [23,24]. In line with this theory, satisfaction with ABW environments appeared to be related to individual differences (i.e., strength of need for privacy, need for relatedness, and age), suggesting that establishing needs-supply fit is important for optimizing satisfaction with the work environment. In addition, our findings regarding job characteristics (i.e., job autonomy, social interaction, and internal mobility) suggest that, in close analogy with PE fit, Job-Environment fit (JE fit) deserves attention as well: job characteristics may influence a person’s needs. Thus, PE fit theory seems to provide a useful framework for further elaborating our understanding of the complex relationships between individual differences among workers, their various job characteristics, and their work environments. At the same time, advanced knowledge about these relationships may contribute to the further development of PE fit theory. In particular, this may broaden the scope of PE fit theory, by acknowledging how specific perceived qualities of the physical work environment (e.g., availability, functionality, comfort, and aesthetic quality of different types of settings) may be instrumental for creating PE fit.

With regard to job characteristics, our findings confirm the model developed by Duffy & Powel [27], which assumes that ABW environments are suitable specifically for work that is highly interactive and highly autonomous. This also implies that they are less suitable for other types of jobs, which may help explain why satisfaction with ABW environments often falls short of expectations.

Probably the most important implication for practice is that managers and workplace professionals should seriously consider individual differences among workers. Our findings underline the importance of analyzing psychological needs, job characteristics, and demographic variables within the population of workers before designing a new work environment for them. Specifically, need for privacy should be examined as this factor may indicate if and how an ABW environment might be successfully implemented. In general, it is advisable to create a work environment and an associated work culture that allow for different work styles in alignment with different needs strengths, job characteristics, and demographic variables. For example, some workers may want to switch between different workstations in quiet and busy zones, while others may always need a distraction-free workstation at the office, and still others may prefer to use their home office for individual tasks and attend the office exclusively for social interaction and collaborative work.

To enhance satisfaction with ABW environments in practice, it seems of utmost importance to increase experienced levels of privacy, especially for workers high in need for privacy and for older workers. Activity settings that are intended to be used for concentration work deserve special attention, as these should be sufficiently available and tailored to specific person-related and job-related needs. In addition, attention should be paid to minimizing psychological, social, and practical barriers that may prevent workers from using these activity settings when they need to concentrate. It is advisable to consider the privacy offered in other activity settings as well, especially in the main area, which usually has an open-plan layout. Here, the experienced level of privacy may be increased through acoustic measures (e.g., sound-absorbing materials), layout choices (e.g., dividers and distance between workstations), provision of back-up spaces for noise-producing activities (e.g., phone calls, informal meetings), and implementing behavioral rules (e.g., leave the area to make a phone call). For workers high in need for privacy, workstations in (private or shared) closed rooms may be preferred as main workstations instead of workstations in an open-plan layout. In addition, increasing the autonomy of workers with regard to the use of alternative locations (e.g., home office, co-working space) when they need a higher level of privacy than the ABW environment at the office provides, may help resolve privacy issues. Our findings underline the importance of taking an integrated approach, recognizing the interplay between the aforementioned physical and psychosocial factors. The typical ABW solution of providing a number of quiet back-up spaces does not seem sufficient to satisfy all workers.

The share of older workers will grow in many organizations in the near future, especially in European countries, due to demographic changes and regulations that push retirement age up [55]. The distinct privacy needs of an ageing workforce [54], together with the increasing value of ‘deep work’ (i.e., individual focus work) within the emerging knowledge economy [56], should urge practitioners to effectively resolve privacy issues in ABW environments.

## Supporting information

S1 Appendix Questionnaire. (DOCX)

S2 Appendix Assumptions and diagnostic plots of model residuals. (DOCX)

S1 Table Confidence intervals and *p*-values for the correlations. (DOCX)

S1 Dataset Dataset. (XLSX)

## References

[1] Cushman & Wakefield. (2013). Workplace transformation survey; a global view of workplace change.

[2] Wohlers C., & Hertel G. (2016). Choosing Where to Work at Work–Towards a Theoretical Model of Benefits and Risks of Activity-Based Flexible Offices. Ergonomics. 10.1080/00140139.2016.1188220 27167298

[3] Leesman. (2017). The rise and rise of Activity Based Working. London: Leesman Available from: http://www.leesmanindex.com/The_Rise_and_Rise_of_Activity_Based_Working_Research_book.pdf

[4] Veldhoen E. (2008). The Art of Working. Academic Service.

[5] Jones Lang Lasalle. (2012). Activity based working. Available from: http://www.jll.com.au/australia/en-au/Documents/jll-au-activity-based-working-2012.pdf

[6] Becker F. (1999). Beyond alternative officing: Infrastructure on-demand. Journal of Corporate Real Estate, 1 (2), 154–168.

[7] Harris R. (2015). The changing nature of the workplace and the future of office space. Journal of Property Investment & Finance, 33 (5), 424–435.

[8] Oseland N., & Webber C. (2012). Flexible Working Benefits. Workplace Unlimited.

[9] Van der Voordt T. (2004). Productivity and employee satisfaction in flexible workplaces. Journal of Corporate Real Estate (6:2), 133–148.

[10] Bodin Danielsson C., & Bodin L. (2009). Difference in satisfaction with office environment among employees in different office types. Journal of Architectural & Planning Research, 26 (3), 241–258.

[11] De Been I., & Beijer M. (2014). The influence of office type on satisfaction and perceived productivity support. Journal of Facilities Management (12:2), 142–157.

[12] Carlopio J. (1996). Construct Validity of a Physical Work Environment Satisfaction Questionnaire. Journal of Environmental Psychology, 27, 177–189. 10.1037//1076-8998.1.3.330 9547055

[13] Veitch J., Charles K., Farley K., & Newsham G. (2007). A model of satisfaction with open-plan office conditions: COPE field findings. Journal of Environmental Psychology 27, 177–189.

[14] Rashid M., & Zimring C. (2008). A Review of the Empirical Literature on the Relationships Between Indoor Environment and Stress in Health Care and Office Settings; Problems and Prospects of Sharing Evidence. Environment and Behavior 40 (2), 151–190.

[15] Ipsos. (2016). Engagement and the Global Workplace. Steelcase Inc Available from: https://cdn2.hubspot.net/hubfs/1822507/2016-WPR/EN/2016-WPR-PDF-360FullReport-EN_.pdf

[16] Brill M., & Weidemann S. (2001). Disproving widespread myths about workplace design Bosti Associates. Buffalo: Kimball International.

[17] Kampschroer K., & Heerwagen J. (2005). The strategic workplace: development and evaluation. Building Research & Information, 33 (4), 326–337.

[18] Brunia S., De Been I., & Van der Voordt T. (2016). Accommodating new ways of working: lessons from best practices and worst cases. Journal of Corporate Real Estate 18 (1), 30–47.

[19] Hoendervanger J., De Been I., Van Yperen N., Mobach M., & Albers C. (2016). Flexibility in Use; Switching behaviour and satisfaction in activity-based work environments. Journal of Corporate Real Estate, 18 (1), 48–62.

[20] Seddigh A., Berntson E., Platts L., & Westerlund H. (2016). Does Personality Have a Different Impact on Self-Rated Distraction, Job Satisfaction, and Job Performance in Different Office Types?. PLoS ONE 1–14. 10.1371/journal.pone.0155295 PMC4880328 27223898

[21] Oldham G., & Fried Y. (1987). Employee reactions to workspace characteristics. Journal of Applied Psychology, 72, 75–80.

[22] Maher A., & Von Hippel C. (2005). Individual differences in employee reactions to open-plan offices. Journal of Environmental Psychology, 25, 219–229.

[23] Edwards J., Caplan R., & Harrison R. (1998). Person-environment fit theory: Conceptual foundations, empirical evidence, and directions for future research In Cooper C, & (Ed.), Theories of organizational stress (pp. 28–67). Oxford: Oxford University Press.

[24] Kristof-Brown A., Zimmerman R., & Johnson E. (2005). Consequences of individuals' fit at work; a meta-analysis of person-job, person-organization, person-group and person-supervisor fit. Personnel Psychology, 281–342.

[25] Van Yperen N., Rietzschel E., & De Jonge K. (2014). Blended Working: For Whom It May (Not) Work. PLoS ONE, 9 (7), 1–8. 10.1371/journal.pone.0102921 PMC4102582 25033202

[26] Oldham G. (1988). Effects of Changes in Workspace Partitions and Spatial Density; A Quasi-Experiment. Journal of Applied Psychology, 253–258.

[27] Duffy, F., & Powell, K. (1997). The New Office. Conran Octopus.

[28] Van Koetsveld R., & Kamperman L. (2011). How flexible workplace strategies can be made successful at the operational level. Corporate Real Estate Journal (1:4), 303–319.

[29] Pullen, W. (2014, September). Age, office type, job satisfaction and performance. Work & Place 9–22. Available from: http://workplaceinsight.net/wp-content/uploads/2014/08/Work+Place4mje.pdf

[30] Hackman J., & Lawler E. (1971). Employee reactions to job characteristics. Journal of Applied Psychology 55 (3), 259–286.

[31] McClelland D., & Burnham D. (1976). Power is the great motivator. Harvard Business Review (54), 100–110. 12545928

[32] Van den Broeck A., Vansteenkiste M., De Witte H., Soenens B., & Lens W. (2010). Capturing autonomy, competence, and relatedness at work: Construction and initial validation of the Work-related Basic Need Satisfaction scale. Journal of Occupational and Organizational Psychology (83), 981–1002.

[33] Van Yperen N., & Wörtler B. (2017). Blended working In Hertel G, Stone D, Johnson R, & Passmore J, The Wiley-Blackwell Handbook of The Psychology of the Internet at Work. Chichester: Wiley-Blackwell.

[34] Baumeister R., & Leary M. (1995). The Need to Belong: Desire for Interpersonal Attachments as a Fundamental Human Motivation. Psychological Bulletin, 497–529. 7777651

[35] Slijkhuis, J. (2012). A structured approach to need for structure at work. Dissertation, University of Groningen.

[36] Sundstrom E., Burt R., & Kamp D. (1980). Privacy at Work: Architectural Correlates of Job Satisfaction and Job Performance. Academy of Management Journal, 23 (1), 101–117.

[37] Altman I. (1975). *The environment and social behavior*: *privacy*, *personal space*, *territory and crowding*. Monterey, CA: Brooks/Cole.

[38] Davenport T. (2005). Thinking for a living: how to get better performance and results from knowledge workers. Harvard Business Press.

[39] Hackman J.R. & Oldham G.R. (1980). Work Redesign. Reading, MA: Addison-Wesley.

[40] Appel—Meulenbroek H., De Vries B., & Weggeman M. (2017). Knowledge sharing behavior: the role of spatial design in buildings. Environment and Behavior, 49 (8), 874–903.

[41] Greene C., & Myerson J. (2011). Space for thought: designing for knowledge workers. Facilities (29:1/2), 19–30.

[42] Volker L., & Van der Voordt D. (2005). An integral tool for the diagnostic evaluation of non-territorial offices. Designing Social Innovation, Planning, Building, Evaluating 241–250.

[43] Li K., Hasher L., Jonas D., & Rahhal T. (1998). Distractibility, Circadian Arousal, and Aging: A Boundary Condition? Psychology and aging 13 (4), 574–583. 9883458 10.1037//0882-7974.13.4.574

[44] Horváth J., Czigler I., Birkás E., Winkler I., & Gervai J. (2009). Age-related differences in distraction and reorientation in an auditory task. Neurobiology of Aging, 30 (7), 1157–1172. doi: 10.1016/j.neurobiolaging.2007.10.003 18023507 10.1016/j.neurobiolaging.2007.10.003

[45] Wells M. (2000). Office Clutter or Meaningful Personal Displays: The Role of Office Personalization on Employee and Organizational Well-Being. Journal of Environmental Psychology, 20 (3), 239–255.

[46] Kaarlela-Tuomaala A., Helenius R., Keskinen E., & Hongisto V. (2009). Effects of acoustic environment on work in private office rooms and open-plan offices—longitudinal study during relocation. Ergonomics, 52 (11), 1423–1444. doi: 10.1080/00140130903154579 19851909 10.1080/00140130903154579

[47] Wanous J., Reichers A., & Hurdy M. (1997). Job Satisfaction: How Good Are Single-Item Measures? Journal of Applied Psychology, 82 (2), 247–252. 9109282 10.1037/0021-9010.82.2.247

[48] Thompson, M.M., Naccarato, M.E., Parker, K.C.H., Moskowitz, G.B. (2001). The personal need for structure and personal fear of invalidity measures: Historical perspectives, current applications, and future directions. In: Moskowitz, G.B., editor. Cognitive Social Psychology: The Princeton Symposium on the legacy and future of social cognition. Mahwah, New York: Lawrence Erlbaum. 19–39.

[49] McKechnie G. (1977). The environmental response inventory in application. Environment and Behavior (9/2), 255–276.

[50] Bruni, C., Schultz, P., & Saunders, C. (n.d.). Environmental Response Inventory. Retrieved 12 2013, from CONPSYCHMeasures; Measurement Tools for Environmental Practioners: www.conpsychmeasures.com/conpsychmeasures/measures/ERI/ERI.html

[51] Cohen J. (1992). A power primer. Psychological Bulletin, 112 (1), 155–159. 19565683 10.1037//0033-2909.112.1.155

[52] Schwartz G. (1978). Estimating the Dimension of a Model. The Annals of Statistics (6), 461–464.

[53] Gerdenitsch C., Kubicek B., & Korunka C. (2015). Control in Flexible Working Arrangements; When Freedom becomes Duty. Journal of Personnel Psychology, 14 (2), 61–69.

[54] Myerson J., Bichard J., & Erlich A. (2010). New demographics, new workspace: Office design for the changing workforce. Farnham, UK: Gower Publishing Ltd.

[55] Aiyar, S., Ebeke, C., & Shao, X. (2016). The Impact of Workforce Aging on European Productivity (IMF Working Paper). International Monetary Fund.

[56] Newport C. (2016). Deep Work. New York: Grand Central Publishing.

