# Supplementary material for: Individual differences in satisfaction with activity-based work environments
Source: PLoS One. 2018 Mar 8;13(3):e0193878. doi: 10.1371/journal.pone.0193878 (PMC5843264; doi:10.1371/journal.pone.0193878)
Supplement: S1 Appendix — (DOCX) [file pone.0193878.s001.docx]

**S1 Appendix. Questionnaire**

Question Answer type

1. Gender: male / female
2. Age: years
3. I have freedom to carry out my tasks the way I prefer. five-point scale*
4. My work involves interaction with other people. five-point scale*
5. I work at different places within the office building. five-point scale*
6. Satisfaction with my work environment: grade (1 - 10)
7. I have the need to have a say in determining my activities and tasks. five-point scale*
8. I have the need for rules and guidelines that I can follow. five-point scale*
9. I have the need to determine on my own how to best approach my work. five-point scale*
10. I have the need for complete silence. five-point scale*
11. I have the need to be around people so I do not feel alone. five-point scale*
12. I get irritated when colleagues are noisy. five-point scale*
13. I have the need to feel like I am part of a team or a group. five-point scale*
14. I have the need to hang out with people. five-point scale*
15. I get distracted easily. five-point scale*
16. I have the need for a daily routine. five-point scale*
17. I have the need to know exactly what to expect. five-point scale*
18. I have the need to decide on my own how to go about getting my job done. five-point scale*
19. I have difficulty concentrating when things are noisy. five-point scale*
20. I have the need for freedom to do my work the way I think is best. five-point scale*
21. I have the need to be with other people. five-point scale*
22. I am easily distracted by people moving about. five-point scale*
23. I have the need for order and regularity. five-point scale*

* five-point scale: (1) very strongly, (2) strongly, (3) moderately, (4) slightly, (5) not at all

Relations between questions and variables

- Gender = 1
- Age = 2
- Job autonomy = 3
- Social interaction = 4
- Internal mobility = 5
- Satisfaction with the work environment = 6
- Need for structure = 8 + 16 + 17 + 23
- Need for autonomy = 7 + 9 + 18 + 20
- Need for relatedness = 11 + 13 + 14 + 21
- Need for privacy = 10 + 12 + 15 + 19 +22
