# Supplementary material for: Individual differences in satisfaction with activity-based work environments
Source: PLoS One. 2018 Mar 8;13(3):e0193878. doi: 10.1371/journal.pone.0193878 (PMC5843264; doi:10.1371/journal.pone.0193878)
Supplement: S2 Appendix — (DOCX) [file pone.0193878.s002.docx]

**S2 Appendix. Assumptions and diagnostic plots of model residuals**

The residuals appear to be independently distributed as indicated by a plot of successive residuals displayed in Figure 1. Even though the assumption of uncorrelated errors was rejected by the Durbin-Watson test (*p* = .02), which tests for autocorrelation between the residuals, this might be due to the large sample size as no obvious problems with correlations are indicated by Figure 1. The plot in Figure 1 indicates a single outlier, which gets plotted twice in this plot. This finding might imply that model errors are uncorrelated between employees even though employees were sampled nested within companies. Hence, even though employees are spatially related this did not result in dependence between their data. Furthermore, a QQ-plot of the residuals shows that the model residuals are roughly normally distributed, as can be seen in the upper right plot of Figure 2. The upper left plot of Figure 2 plots the residuals against predicted values, the lower left plot shows square root standardized residuals against predicted values. The plots suggest that residuals appear to exhibit constant variance. Further, the upper left plot suggests that the relationship between the predictors and workplace satisfaction can be modelled in a linear fashion, though an outlier is visible in all plots included in Figure 2 (participant 337). A plot of residuals against leverages, depicted in the lower right of Figure 2, suggests that there are no data points which severely distort the model prediction, while one outlier is visible in the final model (participant 337). This outlier has associated values of one on satisfaction with the work environment (*Cook's distance* = .04). However, a comparison of the final model with an equivalent model with the outlier excluded demonstrated that the outlier did not influence the model in any relevant way. The outlier was included in the final model.


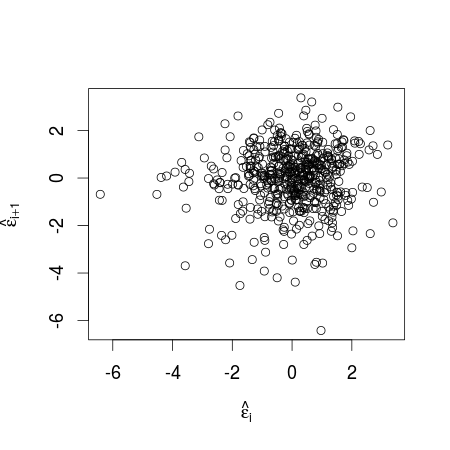


*Figure 1*: Successive Residuals of the final regression model.


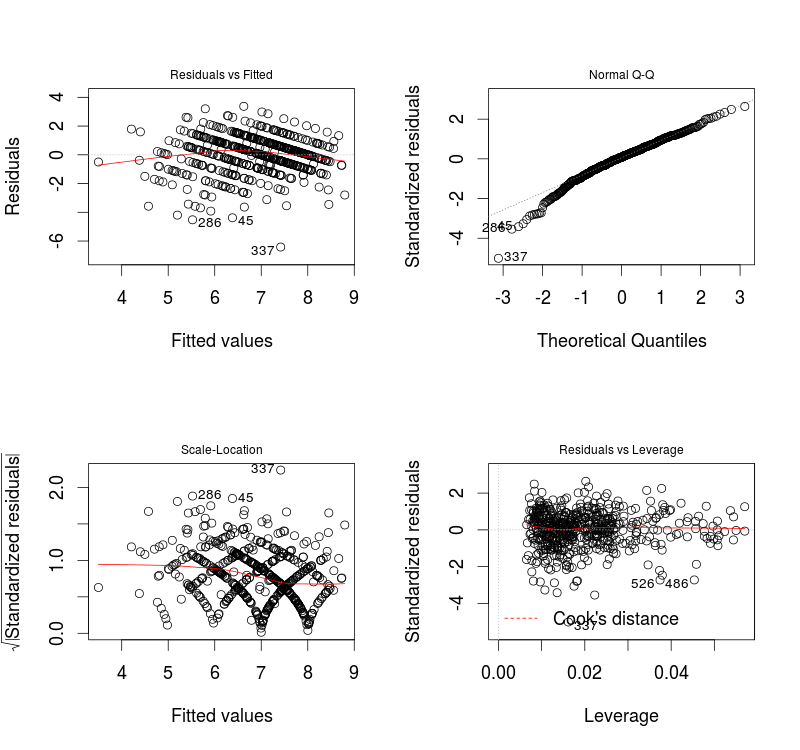


*Figure 2:* Assumption checks of the final linear regression model. The upper left and lower left plot show the residuals and square-root standardized residuals plotted against their predicted values, respectively. The upper right plot depicts a QQ-plot of the residuals. The lower-right plot allows for detection of influential points by plotting residuals against leverage.
