# Supplementary material for: Individual differences in satisfaction with activity-based work environments
Source: PLoS One. 2018 Mar 8;13(3):e0193878. doi: 10.1371/journal.pone.0193878 (PMC5843264; doi:10.1371/journal.pone.0193878)
Supplement: S1 Table — (DOCX) [file pone.0193878.s003.docx]

**S1 Table. Confidence intervals and *p*-values for the correlations**

| Variable | 1. | 2. | 3. | 4. | 5. | 6. | 7. | 8. |
| --- | --- | --- | --- | --- | --- | --- | --- | --- |
| 1. Satisfaction with the work environment | - |  |  |  |  |  |  |  |
| 2. Need for autonomy | -.04 - .13 (.260) | - |  |  |  |  |  |  |
| 3. Need for relatedness | .02 - .19 (.014) | -.04 - .13 (.329) | - |  |  |  |  |  |
| 4. Need for structure | -.15- .02 (.122) | -.34 - -.19 (.000) | .04 - .20 (.005) | - |  |  |  |  |
| 5. Need for privacy | -.46 - -.32 (,000) | -.03 - .14 (.167) | -.21 - -.04 (.003) | .19 - .35 (.000) | - |  |  |  |
| 6. Age | -.29 - -.13 (.000) | -.13 - .04 (.342) | -.30 - -.14 (.000) | -.15 - .02 (.116) | -.09 - .07 (.801) | - |  |  |
| 7. Job autonomy | .13 - .29 (.000) | .44 - .57 (.000) | -.05 - .12 (.395) | -.31 - -.15 (.000) | -.17 - -.01 (.031) | -.09 - .07 (.813) | - |  |
| 8. Social interaction | .13 - .29 (.000). | .33 – .47 (.000) | .09 - .26 (.000) | -.27 - -.11 (.000) | -.23 - -.07 (.000) | -.20 - -.03 (.007) | .35 - .48 (.000) | - |
| 9. Internal mobility | .06 - .22 (.001) | .13 – .29 (.000) | -.05 - .11 (.471) | -.31 - -.16 (.000) | -.10 - .07 (.769) | -.27 - -.11 (.000) | .17 - .32 (.000) | .19 - .35 (.000) |

S1 Table provides 95% confidence intervals and exact *p*-values for the correlations.
